# Supplementary figures and images for: Mapping the hidden diversity of the Geophagus sensu stricto species group (Cichlidae: Geophagini) from the Amazon basin
Source: PeerJ. 2021 Nov 30;9:e12443. doi: 10.7717/peerj.12443 (PMC8641480; doi:10.7717/peerj.12443)

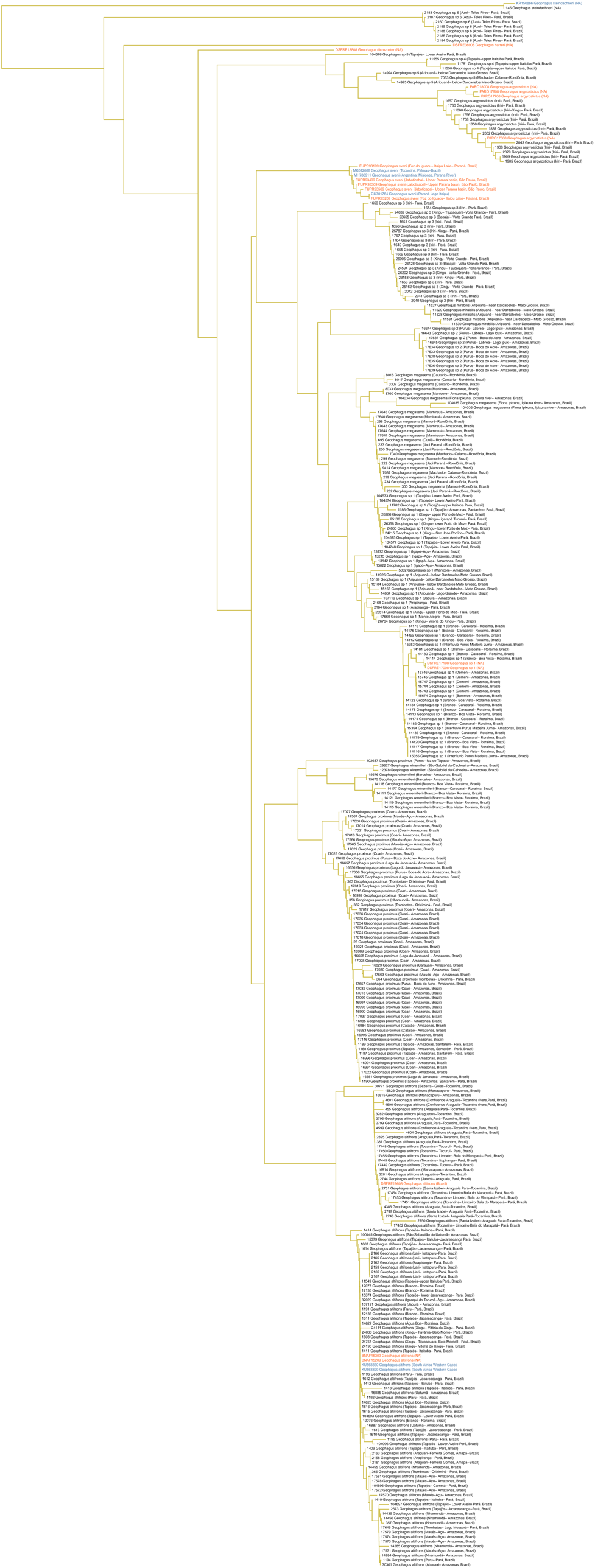

Supplement: Supplemental Information 2 [file peerj-09-12443-s002.pdf]

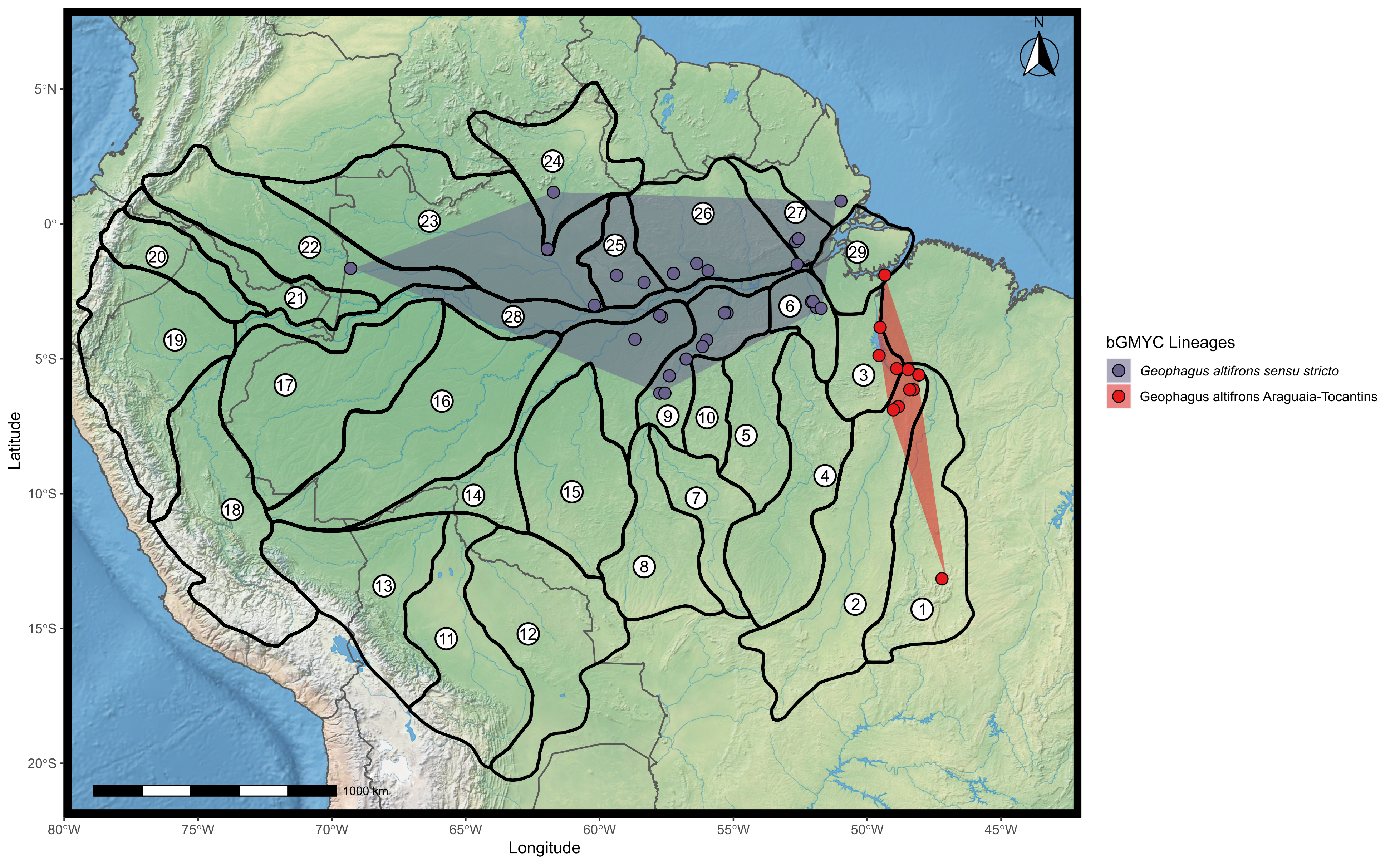

Supplement: Supplemental Information 3 — The points were plotted on the shape of the biogeographic units proposed for fish in the Amazon basin, provided by Dagosta & De Pinna (2017). The map was constructed in R 4.0.0 using packages ‘ggspatial’, ‘raster’, ‘rgdal’, ‘rnaturalearth’, and ‘tidyverse’. The final image was edited in Inkscape. [file peerj-09-12443-s003.png]

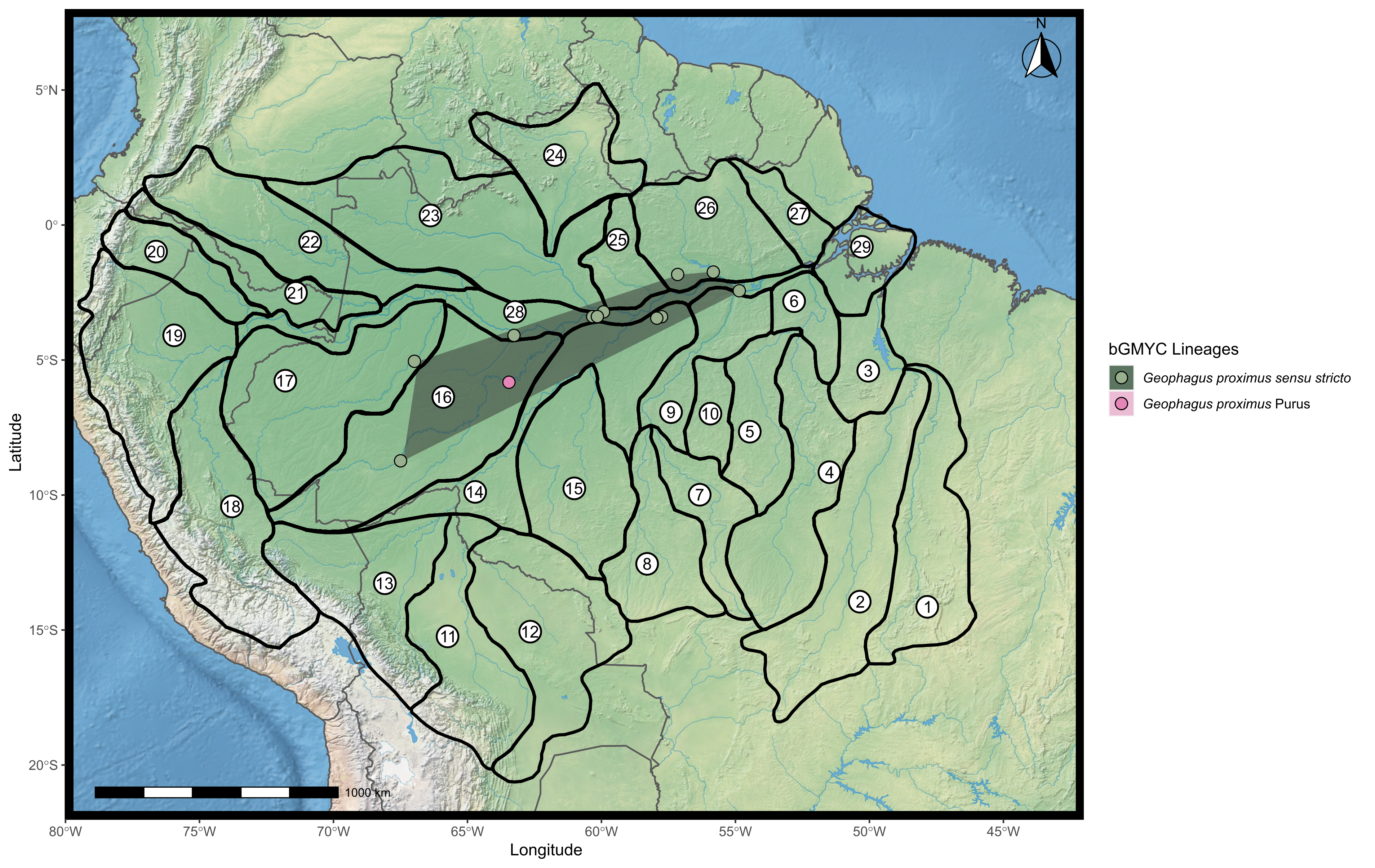

Supplement: Supplemental Information 4 — Geophagus proximus Purus lineage was represented by only one point and it is not possible to construct the minimum polygon. The points were plotted on the shape of the biogeographic units proposed for fish in the Amazon basin, provided by Dagosta & De Pinna (2017). The map was constructed in R 4.0.0 using packages ‘ggspatial’, ‘raster’, ‘rgdal’, ‘rnaturalearth’, and ‘tidyverse’. The final image was edited in Inkscape. [file peerj-09-12443-s004.png]

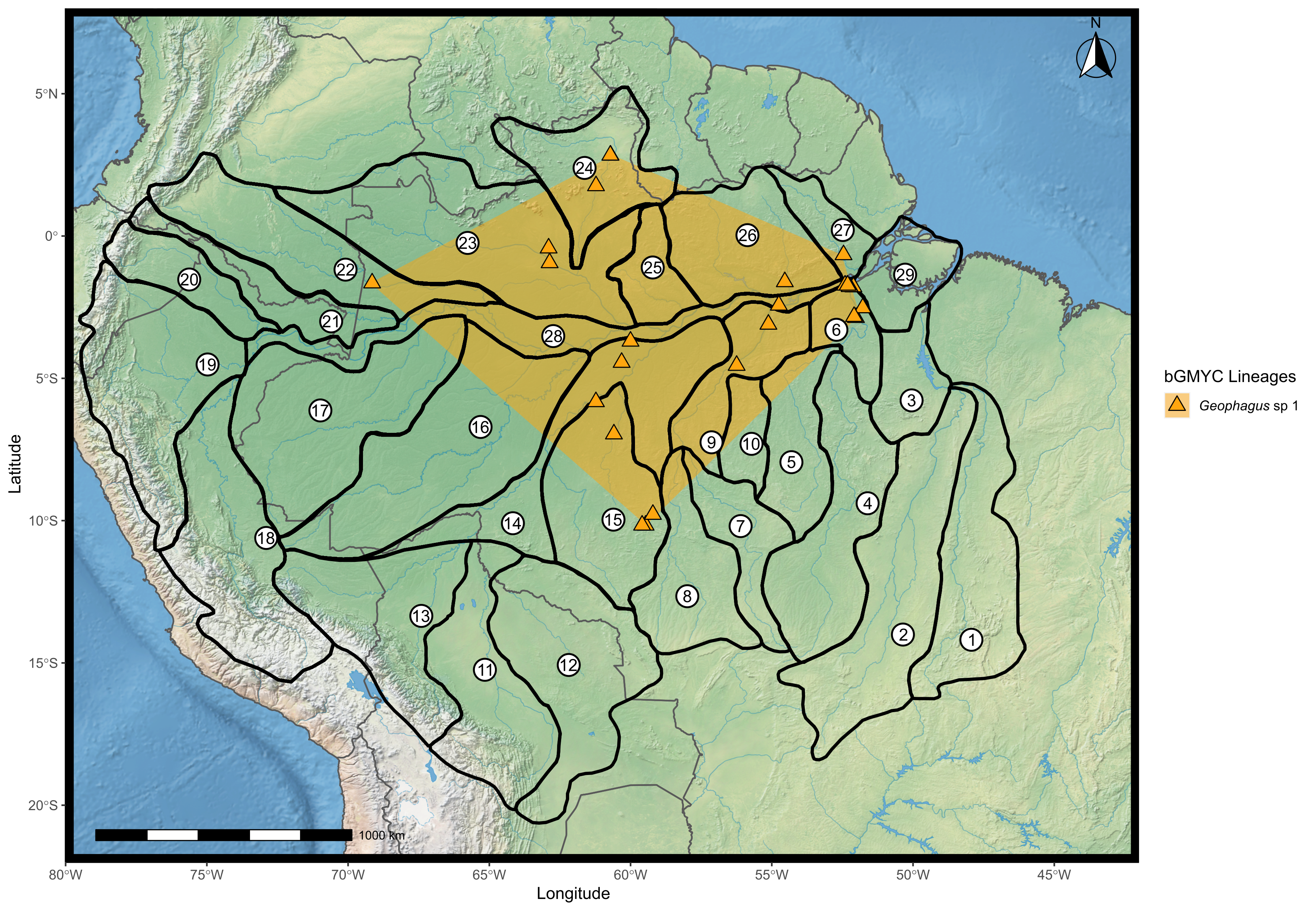

Supplement: Supplemental Information 5 — The points were plotted on the shape of the biogeographic units proposed for fish in the Amazon basin, provided by Dagosta & De Pinna (2017). The map was constructed in R 4.0.0 using packages ‘ggspatial’, ‘raster’, ‘rgdal’, ‘rnaturalearth’, and ‘tidyverse’. The final image was edited in Inkscape. [file peerj-09-12443-s005.png]

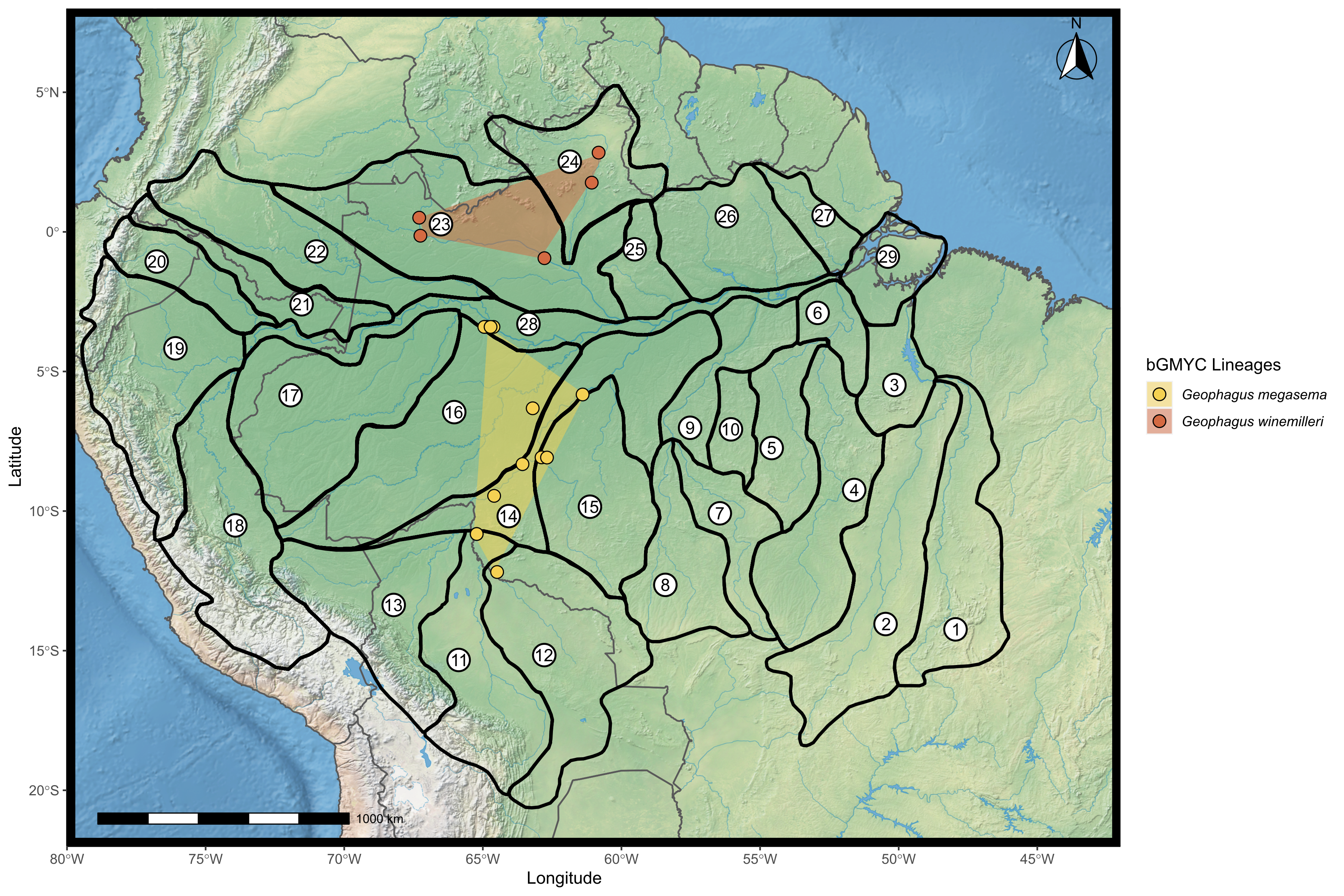

Supplement: Supplemental Information 6 — The points were plotted on the shape of the biogeographic units proposed for fish in the Amazon basin, provided by Dagosta & De Pinna (2017). The map was constructed in R 4.0.0 using packages ‘ggspatial’, ‘raster’, ‘rgdal’, ‘rnaturalearth’, and ‘tidyverse’. The final image was edited in Inkscape. [file peerj-09-12443-s006.png]

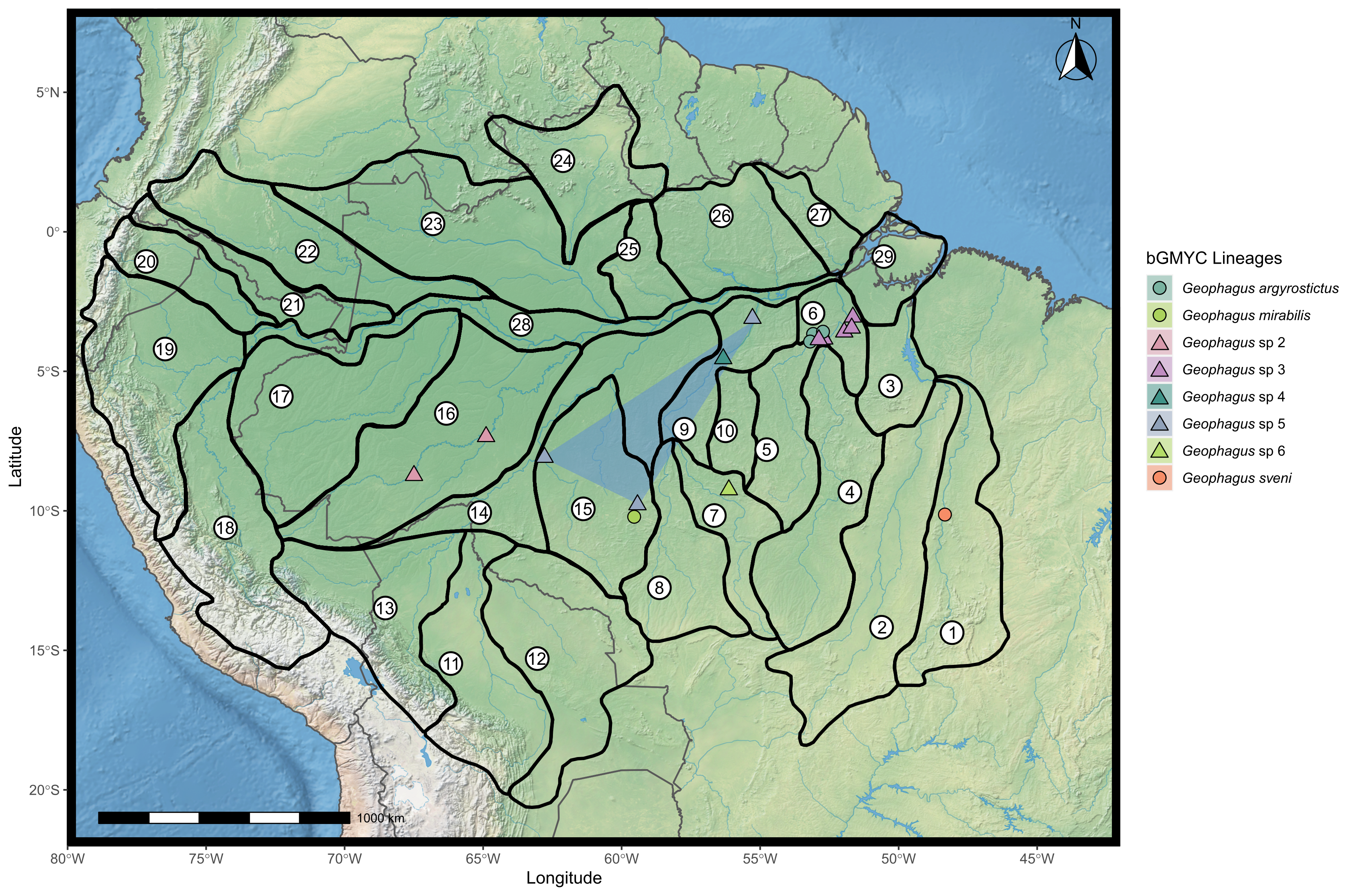

Supplement: Supplemental Information 7 — The points were plotted on the shape of the biogeographic units proposed for fish in the Amazon basin, provided by Dagosta & De Pinna (2017). The map was constructed in R 4.0.0 using packages ‘ggspatial’, ‘raster’, ‘rgdal’, ‘rnaturalearth’, and ‘tidyverse’. The final image was edited in Inkscape. [file peerj-09-12443-s007.png]
